# ImgLib2—generic image processing in Java

**DOI:** 10.1093/bioinformatics/bts543

**Published:** 2012-09-08

**Authors:** Tobias Pietzsch, Stephan Preibisch, Pavel Tomančák, Stephan Saalfeld

**Affiliations:** ^1^Max Planck Institute of Molecular Cell Biology and Genetics, 01307 Dresden, Germany and ^2^Janelia Farm Research Campus, Howard Hughes Medical Institute, Ashburn, VA 20147, USA

## Abstract

**Summary**: ImgLib2 is an open-source Java library for *n*-dimensional data representation and manipulation with focus on image processing. It aims at minimizing code duplication by cleanly separating pixel-algebra, data access and data representation in memory. Algorithms can be implemented for classes of pixel types and generic access patterns by which they become independent of the specific dimensionality, pixel type and data representation. ImgLib2 illustrates that an elegant high-level programming interface can be achieved without sacrificing performance. It provides efficient implementations of common data types, storage layouts and algorithms. It is the data model underlying ImageJ2, the KNIME Image Processing toolbox and an increasing number of Fiji-Plugins.

**Availability**: ImgLib2 is licensed under BSD. Documentation and source code are available at http://imglib2.net and in a public repository at https://github.com/imagej/imglib.

**Supplementary Information:** Supplementary data are available at *Bioinformatics* Online.

**Contact**: saalfeld@mpi-cbg.de

## 1 INTRODUCTION

Many algorithmic concepts from computer vision and image processing are applicable to the analysis of biological image data. However, re-using existing code is often difficult because it is implemented for a specific data type, limited image size or fixed number of dimensions, e.g. small 2d grayscale images. Biological imaging techniques generate images of varying dimensionality and a multitude of sample types (e.g. wavelength, frequency spectra, diffusion tensors) with varying precision. Improvements in imaging speed and resolution result in gigantic datasets that require well-designed strategies for data handling (e.g. tiled or compressed storage, streaming access). Writing code that is re-usable across many combinations of dimensionality, sample type and storage strategy is challenging and requires an appropriate abstraction layer.

We present ImgLib2, an open-source image processing framework that achieves code re-usability through a generic interface architecture that abstracts from dimensionality, sample type and storage strategy. It is highly extensible, providing developers with great flexibility in adding new sample types and image representations that will seamlessly work with existing algorithms, and vice versa. ImgLib2 shares basic concepts with the C++ frameworks ITK (Yoo *et al.*, 2002) and Vigra (Köthe, 2000) for *n*-dimensional, generic image processing. It is the first framework that introduces generic programming to the Java image processing community (Preibisch *et al.*, 2010). We chose Java for its simplicity and wide acceptance among biological researchers due to the popular image processing toolbox ImageJ (Rasband, 2012).

## 2 ARCHITECTURE

The ImgLib2 core design is based on three main concepts: *Accessibles* (i.e. images), *Accessors* and *Types*. We define an image as any mapping from a subset of *n*-dimensional Euclidean coordinate space to a generic pixel value type. Image properties are expressed by *Accessible* interfaces: coordinates can be either integer or real-valued, the coordinate domain can be either bounded or infinite, the image may support random access at arbitrary coordinates and/or iteration of all samples. Consider a conventional pixel image. It comprises samples of a specific value type in bounded *n*-dimensional space, arranged on an integer grid and is both random-accessible (at arbitrary integer coordinates) and iterable. Importantly, ImgLib2 supports concepts beyond the conventional pixel image, e.g. infinite, procedurally generated images or continuous images interpolated from sparsely sampled data.

Access to sample (pixel) values and coordinates is provided by *Accessor* interfaces. These exist in variants for integer and real coordinates, as well as iterating and random access. For iterating accessors, iteration order is subject to implementation, specialized for each memory layout to minimize access time.

Accessors provide value access via *Types*. ImgLib2 has a hierarchy of *Type* interfaces that describe algebraic properties of families of concrete types. Examples are *Comparable* types or *NumericTypes* that support basic arithmetic operations (+,−,*,/).

Access patterns and type properties allow fine-grained specification of algorithmic requirements. An algorithm that is built using appropriate interfaces applies to any specific image implementing those interfaces. Re-usability of algorithms is maximized by specifying them for the minimal set of required properties. Consider, for example, summing all pixel values in an image. This can be implemented in two lines of Java code for, e.g. a gray-level image stored as a byte[] array. However, it has to be re-implemented, over and over, for every combination of data type, dimensionality and storage layout. Using ImgLib2, this can be written generically as

for (T value : image) sum.add(value);

where we specify that image implements *Iterable*
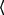
*T*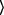 and that *T* extends *NumericType*
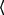
*T*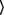. The same code handles all pixel images with appropriate value type, virtual views into such images, sparsely sampled datasets, procedural images, etc.

In Java, this level of generality requires pixels to be objects. Storing simple pixel values (e.g. bytes) as individual objects, however, comes with significant memory overhead. Conversely, creating new objects per pixel access introduces significant runtime overhead and triggers frequent garbage collection. Both approaches do not scale well with large images. To address this issue, ImgLib2 uses *proxy types* to access pixel data that can be mapped into Java primitive type arrays (byte[], float[], etc.). In this way, an accessor can re-use one proxy instance for all pixel accesses. In the above example, a proxy of type *T* is instantiated once and then re-used in every iteration, changing only internal state. This virtualization pattern has no performance overhead compared with direct array access, thanks to the optimizations performed by Java’s just-in-time (JIT) compiler.

**Fig. 1.**
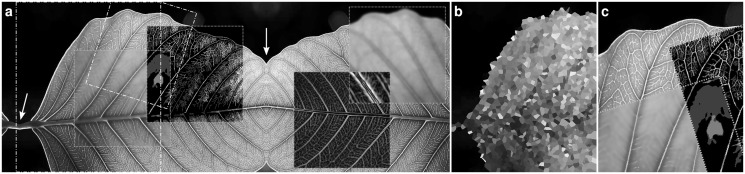
Visualizes exemplarily the capabilities of ImgLib2. (**a**) shows an image, virtually extended by a mirroring strategy, arrows mark the original image boundaries. Four algorithms were applied to sub-image views: (from left to right) anisotropic diffusion, maximally stable extremal regions, Sobel filtering, Gaussian convolution. (**b**) shows an extrapolation of sparse data where 2,000 points were randomly sampled from the larger area indicated in (a). (**c**) shows an interpolated and affine transformed view of the smaller tilted area indicated in (a)

## 3 IMPLEMENTATION

ImgLib2 incorporates common value types (*BitType*, *UnsignedByteType*, *ARGBType*, *ComplexFloatType*, *etc*.) efficiently implemented as proxies that map into Java primitive type arrays. Various implementations for pixel data in a discrete *n*-dimensional grid (conventional pixel images) are provided: *ListImg* stores pixels as individual object instances and thus supports arbitrary value types, but does not scale to large numbers of pixels. *ArrayImg* maps proxy types into a single primitive type array, providing optimal performance and memory efficiency. However, Java arrays are limited to a size of 2^31^ (e.g. a square 2d image with maximally 46,340 px side length) which is easily exceeded in today’s microscopy recordings. *CellImg* splits the coordinate domain into an *n*-dimensional grid of cells, each mapping into one primitive type array. This enables significantly larger images (2^62^ px) at slightly reduced performance. In generic code we can transparently switch between image implementations using image factories. This allows performance tuning for specific datasets without any modification to the algorithm implementation.

We compared the performance of ImgLib2 generic code and special purpose (fixed dimensionality and value type) implementations for Java primitive type arrays and ImageJ (Supplementary Table S1). For simple per-pixel operations, generic ImgLib2 code achieves 100% of the performance of special purpose implementations using native arrays. For a more complex operation involving an inner loop over the unknown number of dimensions, the ImgLib2 code was on average 1.6× slower than native arrays (1.5× slower than ImageJ). We consider this a reasonable abstraction penalty as the ImgLib2 code supports any dimensionality, image and value type. In contrast, native arrays and ImageJ images require specialized implementations for each supported dimensionality and value type. For the cases tested in our benchmark, this amounts to an order of magnitude increase in lines of code. Even so, only ImgLib2 is able to handle all test cases due to dimensionality and image size limits of both ImageJ and primitive type arrays.

ImgLib2 permits virtualization of sample access. We use this for accessors that perform on-the-fly coordinate and value transformations without copying the underlying data. The *Views* framework creates accessibles that provide coordinate-transforming accessors. Integer coordinate transformations include slicing, windowing, axes permutations and 90° rotations. Consecutive transformations are reduced and simplified, yielding accessors with optimal performance. For real coordinates we support *n*-dimensional affine transformations. Interpolating and rasterizing views convert between discrete and continuous coordinate spaces. Finally, some algorithms (e.g. convolution) require access to pixels outside of the image which are usually created by padding or mirroring. This is achieved by extending views, whose accessors generate outside values on demand. Note, that views may be cascaded and act both as input and output for pixel processing. Similarly, the *Converters* framework realizes transparent transformation of values. For instance, a *FloatType* image can be addressed as *ByteType* using an arbitrary mapping function.

ImgLib2 uses Bio-Formats (Linkert *et al.*, 2010) to read and write a large number of image file formats. Interoperability with ImageJ is provided by non-copying wrappers of ImageJ data structures as ImgLib2 accessibles and vice versa. This makes it straightforward to integrate ImgLib2 into existing ImageJ-based processing pipelines. Light-weight wrappers for other data models are easy to implement and currently exist for Java AWT *BufferedImage*, Java primitive type arrays and remotely stored image stacks (Saalfeld *et al.*, 2009). ImgLib2 comprises a growing collection of generic algorithms that are fundamental building blocks for *n*-dimensional image analysis: the Fast Fourier Transform can be used for tomography reconstruction, pattern detection or (de-)convolution; sub-pixel edge detection (Devernay, 1995), component trees (Nistér and Stewénius, 2008) and automatically detected interest-points (e.g. DoG and MSER; Lindeberg, 1998; Matas *et al.*, 2002) are important tools for image segmentation, image registration and tracking; *k*-d trees enable fast *n*-dimensional search.

Sparsely and irregularly sampled data are supported, stored either as a sample list or in a *k*-d tree. Both implement interfaces for nearest-neighbor search, allowing extrapolation of sparse data into a continuous image. Sparsely sampled data, interpolation, extension, coordinate transformation and several algorithms are illustrated in Figure 1.

## 4 DISCUSSION

ImgLib2 is an open-source image processing framework that increases code re-usability by promoting generic implementations. It provides an abstraction layer that focuses on flexible and efficient image storage and access. The core paradigm is a clean separation of pixel algebra (how sample values are manipulated), data access (how sample coordinates are traversed) and data representation (how the samples are stored, laid out in memory or paged to disc). ImgLib2 relies on virtual access to both sample values and coordinates, facilitating parallelizability and extensibility.

ImgLib2 aims to connect software projects through an interface design that is easily adapted to existing data structures. ImgLib2 is the first image processing library available for Java that combines a flexible high-level programming interface with optimal performance. It enables developers of bioimage analysis software to focus on the design of complex algorithms instead of data management. Conversely, software engineers can develop efficient infrastructure without interfering with algorithm design. This becomes particularly interesting in the emerging field of bioimage informatics that is coping with the enormous amount of *n*-dimensional image data generated by recent developments in microscopy. Consequently, ImgLib2 is already being used by several high-profile projects of the Java bioimaging community (Berthold *et al.*, 2009; Rueden *et al.*, 2010; Schindelin *et al.*, 2012). It is easily integrated into other projects providing an ideal basis for sharing interoperable, generic algorithms.

## Supplementary Material

Supplementary Data
