# Supplementary material for: ImgLib2—generic image processing in Java
Source: Bioinformatics. 2012 Sep 8;28(22):3009–11. doi: 10.1093/bioinformatics/bts543 (PMC3496339; doi:10.1093/bioinformatics/bts543)
Supplement: Supplementary Data [file supp_28_22_3009__index.html]

ImgLib2 – Generic Image Processing in Java — ImgLib2—generic image processing in Java — Supplementary Data 

# ImgLib2—generic image processing in Java

## Supplementary Data

files

**Files in this Data Supplement:**

- Supplementary Data - pdf file
- Supplementary Data - avi file
- Supplementary Data - zip file
