# Supplementary material for: ImgLib2—generic image processing in Java
Source: Bioinformatics. 2012 Sep 8;28(22):3009–11. doi: 10.1093/bioinformatics/bts543 (PMC3496339; doi:10.1093/bioinformatics/bts543)
Supplement: Supplementary Data [file supp_bts543_supplementary_methods.pdf]

ImgLib2 – Supplementary Methods

Tobias Pietzsch*, Stephan Preibisch*, Pavel Tomančák, Stephan Saalfeld*†

1. IMGLIB2 DOCUMENTATION

Extensive documentation of ImgLib2 is available via http://imglib2.net. This central webpage is hosted on the fiji-wiki and currently refers to the documentation, a comprehensive collection of examples, the Java-docs and the source-code. The documentation is based on an interactive wikipedia-style page that can be edited and further extended in the future.

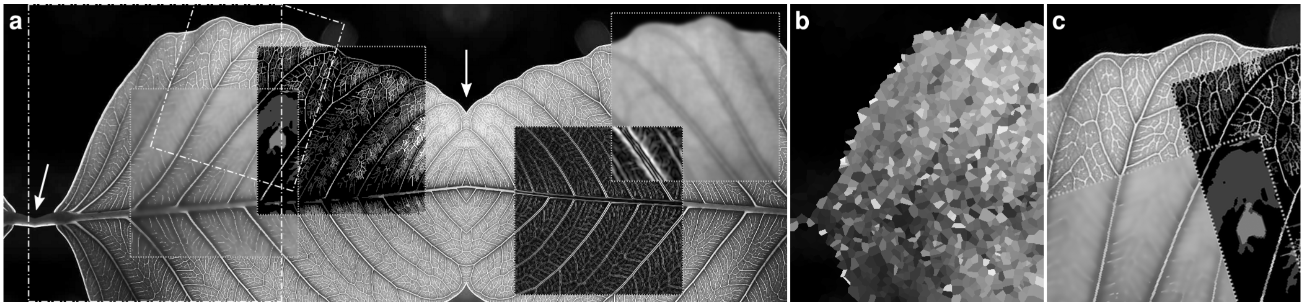

Figure 1. visualizes exemplarily the capabilities of ImgLib2. **(a)** shows an image, virtually extended by a mirroring strategy, arrows mark the original image boundaries. Four algorithms were applied to sub-image views: (from left to right) anisotropic diffusion, maximally stable extremal regions, Sobel filtering, Gaussian convolution. **(b)** shows an extrapolation of sparse data where 2000 points were randomly sampled from the larger area indicated in (a). **(c)** shows an interpolated and affine transformed view of the smaller tilted area indicated in (a).

2. SOURCE CODE FOR FIGURE

In the following we give the code that generates Supplementary Figure 1. We only omit the source code of helper functions for drawing the overlaid boxes and visualizing maximally stable extremal regions. These missing bits are provided in the supplementary `sourcecode.zip`. In the following we walk through the `main` method of `Figure.java` wich loads an input image, applies operators to sub-regions and assembles the result. We show intermediate results after each step. Application of operators is done in helper methods which we will discuss subsequently.

2.1 Main method

```
package figures;

import ...

public class Figure
{
  public static void main(final String[] args)
      throws ImgIOException, IncompatibleTypeException
  {
    final ImgFactory<FloatType> factory = getImgFactory(new FloatType());

    // open source image using an ImgOpener
```

*equal contribution
†to whom correspondence should be addressed

```java
final Img<FloatType> img =
    new ImgOpener().openImg("src/resources/Leaf.tif", factory, new FloatType());
```

This reads the image "Leaf.tif" from disk into an *Img⟨FloatType⟩ img*. The storage strategy of *img* is specified by the *factory*. Image factories throughout the example are created by the `getImgFactory` method which we will discuss below. The *img* looks like this:

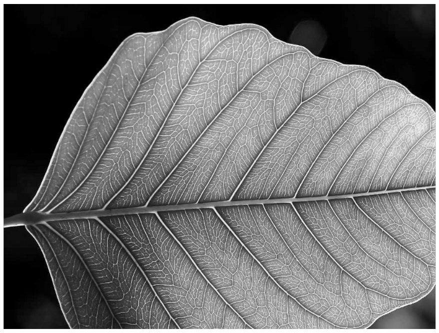

```java
// source view is an interval on the infinitely extended input image
final Interval sourceInterval = Intervals.createMinSize(-50, 0, 2092, 480);
final RandomAccessibleInterval<FloatType> sourceView =
    Views.offsetInterval(Views.extendMirrorSingle(img), sourceInterval);
```

Here we extend *img* to infinity using a mirroring strategy and then restrict this infinite image to the interval starting at $(-50, 0)$ with size $(2092, 480)$. These operations are performed using ImgLib2's Views framework. The resulting views are light-weight objects that perform transformation transparently. The original data is not copied or modified. Transformations are performed on demand, only when pixels are accessed. The following *sourceView* is defined in this way:

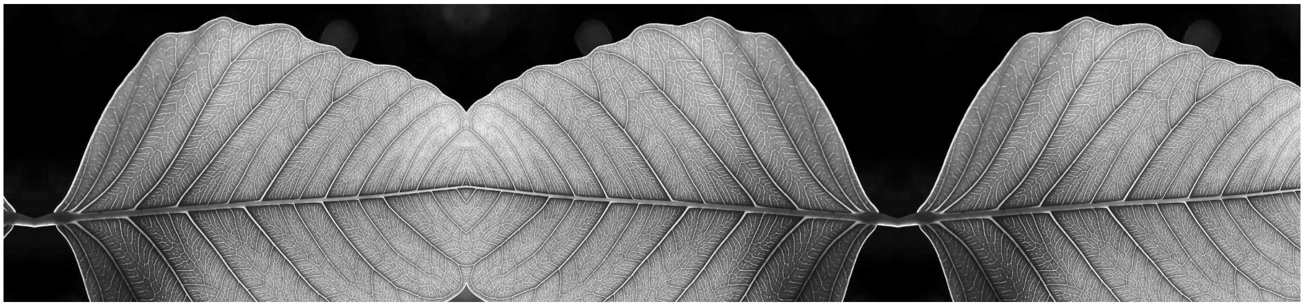

```java
// copy source view to output image
final Img<FloatType> output = factory.create(sourceInterval, new FloatType());
copy(sourceView, output);
```

We create the *output* image that will contain the assembled figure using the same factory as before. We create it of the same size as *sourceView* before and then copy the contents of *sourceView*.

```java
// interpolated random samples
final Interval sampleInterval = Intervals.createMinSize(40, 0, 409, 480);
final long[] sampleOutputOffset = new long[] {1229, 0};
final ArrayList<RealPoint> points = drawRandomRealPoints(sampleInterval, 2000);
randomSampling(output,
    Views.interval(Views.translate(output, sampleOutputOffset), sampleInterval),
    points);
```

We sample 2000 random coordinates uniformly distributed in the interval starting at $(40, 0)$ with size $(409, 480)$. Then the image (*output*) is sampled at these coordinates. A continuous image is extrapolated from the sparse sample set and a portion of that image is rasterized into the target region. The target region is constructed using Views to translate to position $(1229, 0)$ of the output image and then restrict to the boundaries of the sampling interval. The methods `drawRandomRealPoints` and `randomSampling` are discussed below. After this step, *output* looks like this:

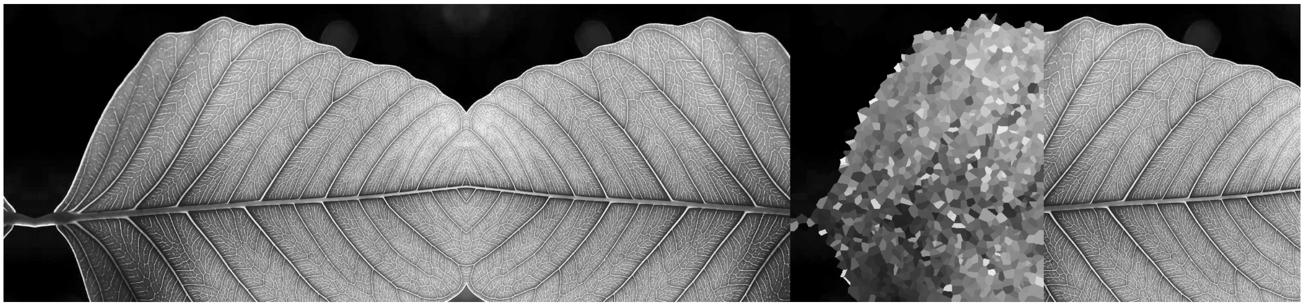

```
76      // anisotropic diffusion
77      final Interval anisoInterval = Intervals.createMinSize(204, 136, 272, 272);
78      anisotropicDiffusionInPlace(Views.interval(output, anisoInterval));
79
80      // MSERs
81      final Interval mserInterval = Intervals.createMinSize(409, 68, 272, 272);
82      visualizeMserTreeInPlace(Views.interval(output, mserInterval));
83
84      // Gauss
85      final Interval gaussInterval = Intervals.createMinSize(980, 14, 272, 272);
86      gaussInPlace(Views.interval(Views.extendMirrorSingle(output), gaussInterval), 3.0);
87
88      // Sobel
89      final Interval sobelInterval = Intervals.createMinSize(824, 197, 272, 272);
90      sobelInPlace(Views.interval(output, sobelInterval));
```

Next we apply different in-place operations to different regions of the *output* image. Regions are again obtained as views. Note that for the Gauss filter we extend the *output* image to infinity before taking the interval view (line 86). This safe-guards against the Gauss filter accessing values outside the image. Note, that this comes essentially for free – the extension will only actually be performed if outside values are required.

```
92      // normalize the output of the Sobel-filter to the value range [0.0, 1.0]
93      Helpers.normInterval(
94          Views.iterable(Views.interval(output, sobelInterval)),
95          new FloatType(0.0f), new FloatType(1.0f));
96
97      // normalize the interval where Sobel-filter is overlapping the Gauss-Filter
98      Helpers.normInterval(
99          Views.iterable(Views.interval(output, Intervals.createMinMax(981, 197, 1095, 284))),
100         new FloatType(0.0f), new FloatType(1.0f));
```

Because the results of the Sobel operator are too dim to be visible, we use a helper function to increase the contrast in the respective intervals. After these steps, *output* looks like this:

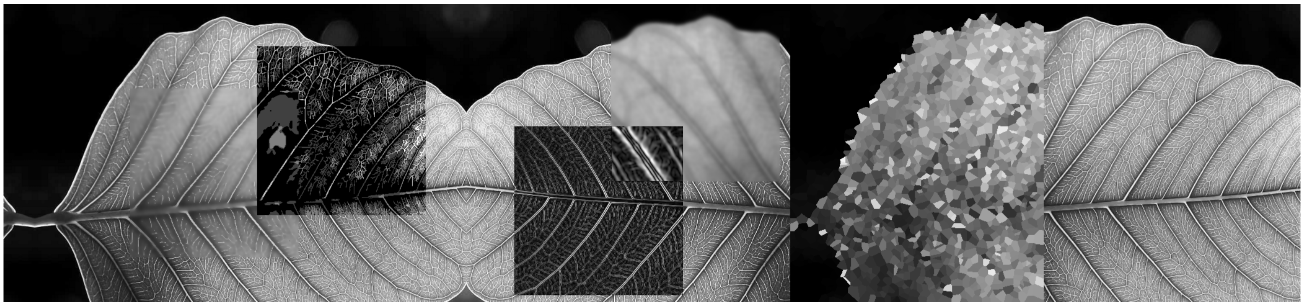

```
102     // draw small boxes with dotted lines
103     final boolean[] dotPattern = new boolean[] { true, false };
104     final FloatType color = new FloatType(0.75f);
105     Helpers.drawBox2D(output, anisoInterval, color, 2, dotPattern);
106     Helpers.drawBox2D(output, mserInterval, color, 2, dotPattern);
107     Helpers.drawBox2D(output, sobelInterval, color, 2, dotPattern);
108     Helpers.drawBox2D(output, gaussInterval, color, 2, dotPattern);
```

We use a helper function to frame the target regions of the above operations with dotted lines.

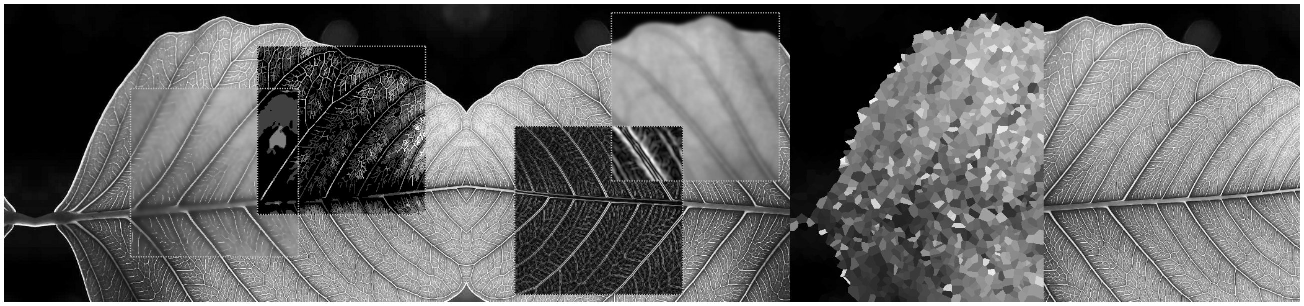

```
110     // apply affine transform
111     final Interval affineOutputInterval = Intervals.createMinSize(1683, 0, 409, 480);
112     final AffineTransform2D affine = new AffineTransform2D();
113     affine.set(1.6241, 0.5024, 1183.1831,
114               -0.5024, 1.6241,  224.7989);
115     affineTransform(
116         Views.extendValue(output, new FloatType(0.0f)),
117         Views.interval(output, affineOutputInterval),
118         affine);
```

We define an affine transformation that should be applied to the image. Then we define an interval in *output* to which the corresponding region of the transformed image is copied. Values outside the original image might be needed to create the transformed image. Therefore we extend the original image to infinity, this time using a constant value (0) for outside pixels (line 116). After this step, *output* looks like this:

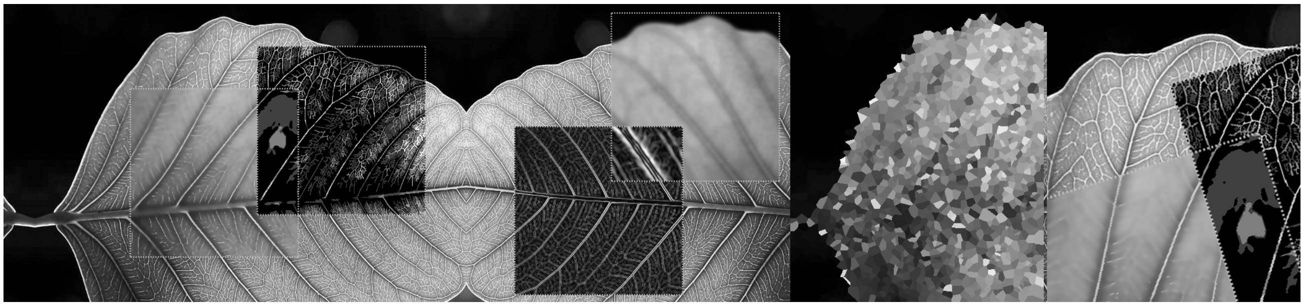

```java
    // draw dashed box for the source regions of sparse sampling and affine transform
    final boolean[] dashPattern =
        new boolean[] {true, true, true, true, true, false, false, true, false, false};
    color.set(1.0f);
    Helpers.drawBox2D(output, sampleInterval, color, 2, dashPattern);
    Helpers.drawAffineBox2D(output, affineOutputInterval, affine.inverse(),
        color, 2, dashPattern);

    // draw separation boxes between sparse sampling and affine transform target regions
    Helpers.fillBox(output, Intervals.createMinSize(1264, 0, 5, 480), color);
    Helpers.fillBox(output, Intervals.createMinSize(1678, 0, 5, 480), color);

    ImageJFunctions.show( output, "output" );
```

Finally, we draw dashed frames around the source regions of the random sampling and affine transform operations and draw separating lines between the respective output regions. We display the result through an ImageJ wrapper.

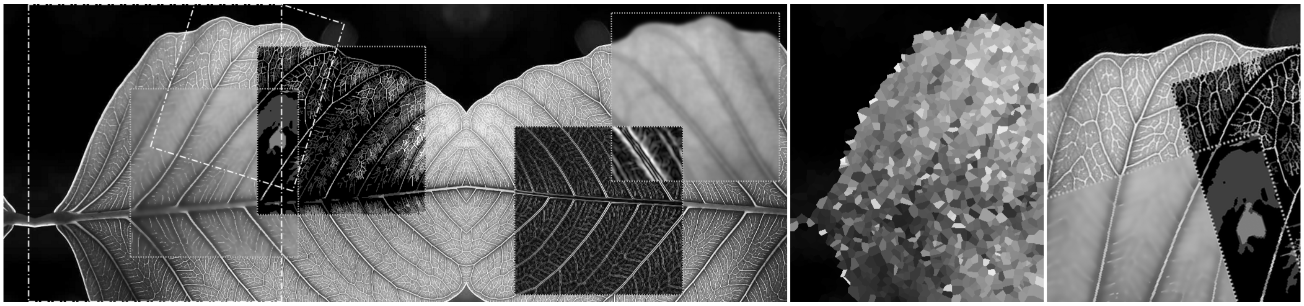

2.2 Auxiliary methods

2.2.1 getImgFactory

```java
  public static <T extends NumericType<T> & NativeType<T>>
      ImgFactory<T> getImgFactory(final T type)
  {
    return new ArrayImgFactory<T>();
  }
```

This is used throughout the example to instantiate image factories. Here we create an *ArrayImgFactory* which is the most efficient, but supports only limited image size. This could be changed to use a different factory throughout the example without changing any of the remaining code. For instance, a *CellImgFactory* could be used if the output image has more than 2G pixels.

2.2.2 copy

```
public static <T extends Type<T>>
    void copy(final RandomAccessible<T> source,
        final RandomAccessibleInterval<T> destination)
{
  final Cursor<T> d = Views.flatIterable(destination).cursor();
  final Cursor<T> s = Views.flatIterable(Views.interval(source, destination)).cursor();
  while (d.hasNext())
    d.next().set(s.next());
}
```

This method copies pixels from *source* to *destination*, such that all pixels of the *destination* interval are filled. This is an example of a truly generic method. It will work for images of every dimensionality because it simply iterates all pixels of the *destination*. It will work for almost any pixel type *T*. We only require that *T* extends $Type\langle T\rangle$ which ensures that the destination pixel values can be set. In the example we apply it to 2D images of *FloatType*.

2.2.3 drawRandomRealPoints

```
public static ArrayList<RealPoint> drawRandomRealPoints(final RealInterval interval,
    final int numPoints)
{
  final ArrayList<RealPoint> points = new ArrayList<RealPoint>(numPoints);

  final Random rnd = new Random(1332441549191l);

  final int n = interval.numDimensions();
  final double[] scale = new double[n];
  final double[] min = new double[n];
  final double[] pos = new double[n];
  interval.realMin(min);
  interval.realMax(scale);
  for (int d = 0; d < n; ++d)
    scale[d] -= min[d];

  for (int i = 0; i < numPoints; ++i) {
    for (int d = 0; d < n; ++d)
      pos[d] = rnd.nextDouble() * scale[d] + min[d];
    points.add(new RealPoint(pos));
  }

  return points;
}
```

This method creates a list of random points uniformly distributed in the specified interval. Note that the dimensionality of the interval determines the dimensionality of the points.

2.2.4 randomSampling

```
public static <T extends NumericType<T>>
    void randomSampling(final RandomAccessible<T> input,
        final RandomAccessibleInterval<T> output,
        final ArrayList<RealPoint> points)
{
```

```java
212       // use linear interpolation to convert the input into a RealRandomAccessible
213       final RealRandomAccessible<T> interpolated =
214           Views.interpolate(input, new NLinearInterpolatorFactory<T>());
215
216       // sample from interpolated image
217       final ArrayList<T> samples = new ArrayList<T>(points.size());
218       final RealRandomAccess<T> a = interpolated.realRandomAccess();
219       for (final RealPoint p : points) {
220         a.setPosition(p);
221         samples.add(a.get().copy());
222       }
223
224       // create a nearest neighbor search backed by a KDTree
225       final NearestNeighborSearch<T> search =
226           new NearestNeighborSearchOnKDTree<T>(new KDTree<T>(samples, points));
227
228       // extrapolate nearest-neighbor search to a RealRandomAccessible
229       final RealRandomAccessible<T> realRandomAccessible =
230           Views.interpolate(search, new NearestNeighborInterpolatorFactory<T>());
231
232       // rasterization turns it into a RandomAccessible for rendering
233       final RandomAccessible<T> rasterized = Views.raster(realRandomAccessible);
234
235       // copy it to output
236       copy(Views.interval(rasterized, output), output);
237     }
```

This method takes a list of coordinates and an input image from which to sample at these coordinates. This results in a sparse sample set, which is extrapolated into a continuous image using nearest-neighbor search. This continuous image is rasterized and copied to the specified output image. Again this is a generic method that works for all storage types and dimensionalities. All pixel types T that extend $NumericType\langle T\rangle$ are supported. *NumericType* is required to support n-linear interpolation of the input image (line 213).

2.2.5 anisotropicDiffusionInPlace

```java
  public static <T extends RealType<T>>
      void anisotropicDiffusionInPlace(final RandomAccessibleInterval<T> data)
  {
    final PeronaMalikAnisotropicDiffusion<T> diffusion =
        new PeronaMalikAnisotropicDiffusion<T>(
            Views.zeroMin(data),
            getImgFactory(new FloatType()),
            0.15,  new PeronaMalikAnisotropicDiffusion.WideRegionEnhancer(0.075));
    for (int i = 0; i < 20; ++i)
      diffusion.process();
  }
```

This method simply applies anisotropic diffusion (from the ImgLib2 algorithms collection) to the specified image *data*. Predefined values are used for the parameters of the algorithm as well as the number of iterations. The restriction of T to $RealType\langle T\rangle$ is required by the algorithm.

2.2.6 visualizeMserTreeInPlace

```java
public static void visualizeMserTreeInPlace(
    final RandomAccessibleInterval<FloatType> data)
{
  final RandomAccessibleInterval<IntType> mserInput = Converters.convert(
      Views.zeroMin(data),
      new Quantizer<FloatType, IntType>(0.0, 1.0, 0, 1000),
      new IntType());
  final MserTree<IntType> treeBrightToDark =
      MserTree.buildMserTree(mserInput, new IntType(10), 20, 10000, 0.4, 0.8, false);

  final FloatType[] colors = new FloatType[] {
      new FloatType(0.25f),
      new FloatType(0.50f),
      new FloatType(0.75f),
      new FloatType(1.00f)};

  for (final FloatType t : Views.iterable(Views.zeroMin(data)))
    t.set(0.0f);

  final VisualizeMserTree<IntType, FloatType> v =
      new VisualizeMserTree<IntType, FloatType>(Views.zeroMin(data), colors);
  v.visualize(treeBrightToDark);
}
```

This method uses an ImgLib2 Converter to quantize values the input *data* to 1000 discrete levels. Then Maximally Stable Extremal Regions (MSER) are extracted from the quantized image using an algorithm from the ImgLib2 algorithms collection. (Quantization is not strictly necessary but speeds up the execution of the algorithm). Predefined values are used for the arameters of the algorithm. Finally, the input *data* is over-written with a visualisation of the extracted MSER regions. Visualisation is done through a helper class that can be found in the supplementary `sourcecode.zip`.

2.2.7 gaussInPlace

```java
public static <T extends RealType<T>>
    void gaussInPlace(final RandomAccessibleInterval<T> data, final double sigma)
{
  final Point min = new Point(data.numDimensions());
  data.min(min);

  final double[] s = new double[data.numDimensions()];
  Arrays.fill(s, sigma);

  // we convolve a region and write it back to the same coordinates
  Gauss.inFloat(s, data, data, data, min, getImgFactory(new FloatType()));
}
```

This method convolves the input image *data* with a n-dimensional Gaussian of the specified standard deviation. Gaussian convolution is an algorithm from the ImgLib2 algorithms collection. The restriction of T to $RealType\langle T\rangle$ is required by the algorithm.

2.2.8 sobelInPlace

```java
public static <T extends RealType<T> & NativeType<T>>
    void sobelInPlace(final RandomAccessibleInterval<T> data)
{
```

```
    final ImgFactory<T> factoryT = getImgFactory(Util.getTypeFromInterval(data));
    final ImgFactory<ComplexFloatType> factoryC = getImgFactory(new ComplexFloatType());

    // create sobel edge filter kernels
    final float[] sX = new float[] {
        -1, 0, 1,
        -2, 0, 2,
        -1, 0, 1};
    final float[] sY = new float[] {
        -1, -2, -1,
         0,  0,  0,
         1,  2,  1};
    final Img<FloatType> sobelX = ArrayImgs.floats(sX, new long[] {3, 3});
    final Img<FloatType> sobelY = ArrayImgs.floats(sY, new long[] {3, 3});

    // apply fourier convolution to convolve input data with kernels
    final Img<T> filterX =
        FourierConvolution.convolve(data, sobelX, factoryT, sobelX.factory(), factoryC);
    final Img<T> filterY =
        FourierConvolution.convolve(data, sobelY, factoryT, sobelY.factory(), factoryC);

    final Cursor<T> cursorSobelX = Views.flatIterable(filterX).cursor();
    final Cursor<T> cursorSobelY = Views.flatIterable(filterY).cursor();
    final Cursor<T> cursorOutput = Views.flatIterable(data).cursor();

    // compute and write gradient for every pixel
    while (cursorSobelX.hasNext()) {
      final double dx = cursorSobelX.next().getRealDouble();
      final double dy = cursorSobelY.next().getRealDouble();
      final double gradient = Math.sqrt(dx * dx + dy * dy);
      cursorOutput.next().setReal(gradient);
    }
  }
```

This method applies the Sobel edge detection operator the input image *data*. The image is convolved with X/Y edge filter kernels in the fourier domain. The gradient is computed from the resulting convolved images and written to *data*.

2.2.9 affineTransform

```
  public static <T extends RealType<T>>
      void affineTransform(final RandomAccessible<T> input,
          final RandomAccessibleInterval<T> output, final AffineTransform2D affine)
  {
    final RealRandomAccessible<T> interpolatedSourceView =
        Views.interpolate(input, new LanczosInterpolatorFactory<T>(0.0, 1.0));
    final RandomAccessible<T> affineTransformedView =
        RealViews.constantAffine(interpolatedSourceView, affine);
    copy(affineTransformedView, output);
  }
```

This method applies an affine transformation to the specified *input* image. First, a continuous image is interpolated from the input (using Lanczos interpolation). Then this is transformed using the specified affine transformation, rasterized, and copied to the specified *output* image.

# dim	size [px]	Java [s]	ImageJ [s]	ArrayImg [s]	CellImg [s]	C++ [s]
**Invert all pixel values**						
1	$100,000,000$	0.080	0.081	0.081	0.39	0.060
2	$8,192^2$	0.054	0.054	0.055	0.26	0.040
2	$50,000^2$	$\times$	$\times$	$\times$	9.79	1.380
3	384^3	0.046	0.049	0.046	0.22	0.030
6	28^6	0.386	$\times$	0.385	1.91	0.265
**Compute the center of mass**						
1	$100,000,000$	1.142	1.250	2.173	2.209	1.140
2	$8,192^2$	1.132	1.323	1.901	1.887	1.050
2	$50,000^2$	$\times$	$\times$	$\times$	70.60	39.15
3	384^3	1.347	1.486	2.008	2.018	1.140
6	28^6	21.45	$\times$	27.72	28.63	16.63

Table 1. Performance of per-pixel operations on ImgLib2 data structures (`ArrayImg` and `CellImg`), native Java arrays and ImageJ's `ImagePlus`. For reference, we include performance of a C++ implementation. The first benchmark (upper half of table) is inverting all pixels in a one- to six-dimensional `float` image. The ImgLib2 implementation achieves native performance with `ArrayImg` while being able to handle images with $> 2^{31}$ pixels using `CellImg` (third row). The second benchmark (lower half of table) is calculating the center-of-mass in a one- to six-dimensional `byte` image. Here, the ImgLib2 code was on average 1.6$\times$ slower than native arrays (1.5$\times$ slower than ImageJ). We consider this to be a reasonable abstraction penalty as the ImgLib2 code supports any dimensionality, image and value type, while native arrays and ImageJ images require specialized implementations for each supported dimensionality and value type. The trade-off in this example is 20 lines of code (loc) for ImgLib2 vs. 260 loc for native *byte* arrays and ImageJ.

3. BENCHMARKS

We compared the performance of ImgLib2 generic code and special purpose (fixed dimensionality and value type) implementations for Java primitive type arrays and ImageJ. For reference, we include a C++ implementation where we use template meta-programming to realize value type and dimensionality independence. In the following we discuss the benchmark tasks and provide the complete source code. The code to set up the input data for the benchmark and collect time measurements is provided in the supplementary `sourcecode.zip`.

We benchmarked two ImgLib2 image types against ImageJ's ImagePlus and primitive Java arrays. Two sets of benchmarks were performed:

Invert image. The task is to invert the value of every pixel in an image. It is sufficient to iterate all pixel values, access to pixel coordinates is not required. This can be implemented concisely and efficiently for ImgLib2, ImageJ, as well as primitive arrays. For primitive arrays, the value type is fixed (we use *float* for the benchmark), whereas both the ImageJ and ImgLib2 implementations provide some degree of type independence. The ImageJ code supports *float* as well as 8 and 16 bit integer value types (by promotion to *float*). The ImgLib2 code supports all *NumericType* value types (because these support the necessary arithmetic operations). The C++ code supports all value types that have a negation operator. In contrast to generic Java code, templated C++ requires the actual type to be fixed at compile time.

Compute center-of-mass of image. The task is to compute a weighted sum of all pixel coordinates in the image, where the weights are the pixel intensities. It is necessary to iterate all pixels and retrieve their values and coordinates. This can be implemented concisely in a dimensionality-independent way for ImgLib2. In

contrast, it is necessary for ImageJ as well as primitive arrays to implement special cases for each supported dimensionality. Our primitive array implementation handles 1D to 6D images, whereas ImageJ supports only 1D to 5D images. The ImgLib2 code handles all dimensionalities. For primitive arrays, the value type is fixed (we use *byte* for the benchmark), whereas both the ImageJ and ImgLib2 implementations provide some degree of type independence. The ImageJ code supports *float* as well as 8 and 16 bit integer value types (by promotion to *float*). The ImgLib2 code supports all *RealType* value types (these support the necessary arithmetic operations). The C++ code supports all value types that can be cast to *double*. In contrast to generic Java code, templated C++ requires the actual type to be fixed at compile time. Additionally, in contrast to ImgLib2 code, the C++ template must be realized for each supported dimensionality at compile time.

The benchmarks were performed on a standard desktop computer with a quad-core Intel® Xeon® CPU with 2.67 GHz and 24 GB of memory, Ubuntu Linux 10.04 LTS installed. The Java™ SE 6 Update 31 runtime environment was used to execute the benchmarks. The C++ versions of the benchmarks were compiled using GCC 4.4.3 with optimization level -O3.

Table 1 shows the median run-times over 20 runs of each benchmark. For simple per-pixel operations (such as the *invert image* benchmark), generic ImgLib2 code achieves 100 % of the performance of special purpose implementations using native arrays. While the first benchmark requires only iteration of all pixel values, the second benchmark requires to access the pixel coordinates as well. For the dimensionality-independent ImgLib2 code, this requires an additional inner loop over the number of dimensions. In this case, the ImgLib2 code was on average 1.6$\times$ slower than native arrays and 1.5$\times$ slower than ImageJ. However, note that the ImgLib2 code supports any dimensionality, image and value type. In contrast, native arrays and ImageJ images require specialized implementations for each supported dimensionality and value type. For the cases tested in our benchmark, this amounts to an order of magnitude increase in lines of code.

Also note, that only ImgLib2 is able to handle the test case of $50,000 \times 50,000$ image. Images of this size no longer fit into a single Java primitive type array, and can therefore not be handled by the Java array and ImageJ code. Additionally, ImageJ is limited to at most 5 dimensions, and is not capable of handling the final 6-dimensional test case.

In the following we give the complete source code for the benchmarks.

3.1 Invert for ImgLib2

```java
package benchmark;

import ...

public class InvertImgLib2Implementation
{
  /**
   * generic implementation for all ImgLib2 {@link IterableInterval}s.
   */
  public final static <T extends NumericType<T>> void invert(final IterableInterval<T> img)
  {
    for (final T t : img)
      t.mul(-1);
  }
}
```

3.2 Invert for ImageJ's ImagePlus

```java
package benchmark;

import ...
```

```java
public class InvertImageJImplementation
{
  /**
   * Invert implementation for ImagePlus. Pixel coordinates are not required so
   * this works for 1D to 5D images.
   */
  public final static void invert(final ImagePlus imp)
  {
    final ImageStack stack = imp.getStack();
    final int numSlices = stack.getSize();
    for (int s = 1; s <= numSlices; ++s) {
      final ImageProcessor ip = stack.getProcessor(s);
      final int size = ip.getPixelCount();
      for (int i = 0; i < size; i++)
        ip.setf(i, -ip.getf(i));
    }
  }
}
```

3.3 Invert for Java float[] array

```java
package benchmark;

public class InvertFloatNativeImplementation
{
  /**
   * Invert implementation for native float[] array. Pixel coordinates are not
   * required so this works for all dimensions.
   */
  public final static void invert(final float[] img)
  {
    for (int i = 0; i < img.length; i++)
      img[i] = -img[i];
  }
}
```

3.4 Invert for C++

```cpp
#ifndef _INVERT_H
#define _INVERT_H

template<class T>
void invert(T* img, unsigned long long size)
{
  for (unsigned long long i = 0; i < size; ++i)
    img[i] = -img[i];
}

#endif
```

3.5 Center-Of-Mass for ImgLib2

```java
package benchmark;

import ...

public class CenterOfMassImgLib2Implementation
{
  /**
   * generic implementation for all ImgLib2 {@link IterableInterval}s.
   */
  public final static <T extends RealType<T>> double[] findCenterOfMass(
      final IterableInterval<T> img)
  {
    final RealSum[] realSums = new RealSum[img.numDimensions()];
    for (int d = 0; d < realSums.length; ++d)
      realSums[d] = new RealSum();
    final RealSum s = new RealSum();
    final Cursor<T> cursor = img.localizingCursor();
    while (cursor.hasNext()) {
      final double w = cursor.next().getRealDouble();
      s.add(w);
      for (int d = 0; d < realSums.length; ++d)
        realSums[d].add(cursor.getDoublePosition(d) * w);
    }

    final double[] centerOfMass = new double[realSums.length];
    final double sum = s.getSum();
    for (int d = 0; d < realSums.length; ++d)
      centerOfMass[d] = realSums[d].getSum() / sum;

    return centerOfMass;
  }
}
```

3.6 Center-Of-Mass for ImageJ's ImagePlus

```java
package benchmark;

import ...

public class CenterOfMassImageJImplementation
{
  /**
   * special purpose implementation for 1D {@link ImagePlus}.
   */
  private final static double[] findCenterOfMass1D(final ImagePlus imp)
  {
    final RealSum centerOfMass0 = new RealSum(),
                  sum = new RealSum();

    final ImageProcessor ip = imp.getProcessor();
    final int size0 = ip.getPixelCount();

    for (int d0 = 0; d0 < size0; ++d0) {
```

```
      final double value = ip.getf(d0);
      centerOfMass0.add(value * d0);
      sum.add(value);
    }
    final double s = sum.getSum();

    return new double[] { centerOfMass0.getSum() / s };
  }

  /**
   * special purpose implementation for 2D {@link ImagePlus}.
   */
  private final static double[] findCenterOfMass2D(final ImagePlus imp)
  {
    final RealSum centerOfMass0 = new RealSum(),
                  centerOfMass1 = new RealSum(),
                  sum = new RealSum();

    final ImageProcessor ip = imp.getProcessor();
    final int size0 = ip.getWidth();
    final int size1 = ip.getHeight();

    int i = 0;
    for (int d1 = 0; d1 < size1; ++d1)
      for (int d0 = 0; d0 < size0; ++d0) {
        final double value = ip.getf(i++);
        centerOfMass0.add(value * d0);
        centerOfMass1.add(value * d1);
        sum.add(value);
      }
    final double s = sum.getSum();

    return new double[] { centerOfMass0.getSum() / s,
                          centerOfMass1.getSum() / s };
  }

  /**
   * special purpose implementation for 3D {@link ImagePlus}.
   */
  private final static double[] findCenterOfMass3D(final ImagePlus imp)
  {
    final RealSum centerOfMass0 = new RealSum(),
                  centerOfMass1 = new RealSum(),
                  centerOfMass2 = new RealSum(),
                  sum = new RealSum();

    final int size0 = imp.getWidth();
    final int size1 = imp.getHeight();
    final int size2 = imp.getNChannels();

    final ImageStack stack = imp.getStack();
    for (int d2 = 0; d2 < size2; ++d2) {
```

```java
      final ImageProcessor ip = stack.getProcessor(imp.getStackIndex(d2 + 1, 1, 1));
      int i = 0;
      for (int d1 = 0; d1 < size1; ++d1)
        for (int d0 = 0; d0 < size0; ++d0) {
          final double value = ip.getf(i++);
          centerOfMass0.add(value * d0);
          centerOfMass1.add(value * d1);
          centerOfMass2.add(value * d2);
          sum.add(value);
        }
    }
    final double s = sum.getSum();

    return new double[] { centerOfMass0.getSum() / s,
                          centerOfMass1.getSum() / s,
                          centerOfMass2.getSum() / s };
  }

  /**
   * special purpose implementation for 4D {@link ImagePlus}.
   */
  private final static double[] findCenterOfMass4D(final ImagePlus imp)
  {
    final RealSum centerOfMass0 = new RealSum(),
                  centerOfMass1 = new RealSum(),
                  centerOfMass2 = new RealSum(),
                  centerOfMass3 = new RealSum(),
                  sum = new RealSum();

    final int size0 = imp.getWidth();
    final int size1 = imp.getHeight();
    final int size2 = imp.getNChannels();
    final int size3 = imp.getNSlices();

    final ImageStack stack = imp.getStack();
    for (int d3 = 0; d3 < size3; ++d3) {
      for (int d2 = 0; d2 < size2; ++d2) {
        final ImageProcessor ip = stack.getProcessor(imp.getStackIndex(d2 + 1, d3 + 1, 1));
        int i = 0;
        for (int d1 = 0; d1 < size1; ++d1)
          for (int d0 = 0; d0 < size0; ++d0) {
            final double value = ip.getf(i++);
            centerOfMass0.add(value * d0);
            centerOfMass1.add(value * d1);
            centerOfMass2.add(value * d2);
            centerOfMass3.add(value * d3);
            sum.add(value);
          }
      }
    }
    final double s = sum.getSum();

```

```java
    return new double[] { centerOfMass0.getSum() / s,
                          centerOfMass1.getSum() / s,
                          centerOfMass2.getSum() / s,
                          centerOfMass3.getSum() / s };
  }

  /**
   * special purpose implementation for 5D {@link ImagePlus}.
   */
  private final static double[] findCenterOfMass5D(final ImagePlus imp)
  {
    final RealSum centerOfMass0 = new RealSum(),
                  centerOfMass1 = new RealSum(),
                  centerOfMass2 = new RealSum(),
                  centerOfMass3 = new RealSum(),
                  centerOfMass4 = new RealSum(),
                  sum = new RealSum();

    final int size0 = imp.getWidth();
    final int size1 = imp.getHeight();
    final int size2 = imp.getNChannels();
    final int size3 = imp.getNSlices();
    final int size4 = imp.getNFrames();

    final ImageStack stack = imp.getStack();
    for (int d4 = 0; d4 < size4; ++d4) {
      for (int d3 = 0; d3 < size3; ++d3) {
        for (int d2 = 0; d2 < size2; ++d2) {
          final ImageProcessor ip = stack.getProcessor(
              imp.getStackIndex(d2 + 1, d3 + 1, d4 + 1));
          int i = 0;
          for (int d1 = 0; d1 < size1; ++d1)
            for (int d0 = 0; d0 < size0; ++d0) {
              final double value = ip.getf(i++);
              centerOfMass0.add(value * d0);
              centerOfMass1.add(value * d1);
              centerOfMass2.add(value * d2);
              centerOfMass3.add(value * d3);
              centerOfMass4.add(value * d4);
              sum.add(value);
            }
        }
      }
    }
    final double s = sum.getSum();

    return new double[] { centerOfMass0.getSum() / s,
                          centerOfMass1.getSum() / s,
                          centerOfMass2.getSum() / s,
                          centerOfMass3.getSum() / s,
                          centerOfMass4.getSum() / s };
  }
```

```java

  /**
   * special purpose implementation for 1D to 5D ImagePlus dispatches to the 1D
   * to 5D versions.
   *
   * @param numDimensions
   *          number of dimensions, because it is impossible to retrieve this
   *          from the ImagePlus.
   */
  public final static double[] findCenterOfMass(final ImagePlus imp, final int numDimensions)
  {
    if (numDimensions == 1)
      return findCenterOfMass1D(imp);
    else if (numDimensions == 2)
      return findCenterOfMass2D(imp);
    else if (numDimensions == 3)
      return findCenterOfMass3D(imp);
    else if (numDimensions == 4)
      return findCenterOfMass4D(imp);
    else if (numDimensions == 5)
      return findCenterOfMass5D(imp);
    else
      throw new IllegalArgumentException("only 1D to 5D images are supported");
  }
}
```

3.7 Center-Of-Mass for Java byte[] array

```java
package benchmark;

import net.imglib2.util.RealSum;

public class CenterOfMassByteNativeImplementation
{
  /**
   * special purpose implementation for native byte[] array 1D.
   */
  private final static double[] findCenterOfMass(final byte[] img, final int size0)
  {
    final RealSum centerOfMass0 = new RealSum(),
                  sum = new RealSum();

    for (int d0 = 0; d0 < size0; ++d0) {
      final double value = img[d0] & 0xff;
      centerOfMass0.add(value * d0);
      sum.add(value);
    }
    final double s = sum.getSum();

    return new double[] { centerOfMass0.getSum() / s };
  }

  /**
```

```java
 * special purpose implementation for native byte[] array 2D.
 */
private final static double[] findCenterOfMass(final byte[] img,
    final int size0, final int size1)
{
  final RealSum centerOfMass0 = new RealSum(),
                centerOfMass1 = new RealSum(),
                sum = new RealSum();

  int i = 0;

  for (int d1 = 0; d1 < size1; ++d1)
    for (int d0 = 0; d0 < size0; ++d0) {
      final double value = img[i++] & 0xff;
      centerOfMass0.add(value * d0);
      centerOfMass1.add(value * d1);
      sum.add(value);
    }
  final double s = sum.getSum();

  return new double[] { centerOfMass0.getSum() / s,
                        centerOfMass1.getSum() / s };
}

/**
 * special purpose implementation for native byte[] array 3D.
 */
private final static double[] findCenterOfMass(final byte[] img,
    final int size0, final int size1, final int size2)
{
  final RealSum centerOfMass0 = new RealSum(),
                centerOfMass1 = new RealSum(),
                centerOfMass2 = new RealSum(),
                sum = new RealSum();

  int i = 0;

  for (int d2 = 0; d2 < size2; ++d2)
    for (int d1 = 0; d1 < size1; ++d1)
      for (int d0 = 0; d0 < size0; ++d0) {
        final double value = img[i++] & 0xff;
        centerOfMass0.add(value * d0);
        centerOfMass1.add(value * d1);
        centerOfMass2.add(value * d2);
        sum.add(value);
      }
  final double s = sum.getSum();

  return new double[]  { centerOfMass0.getSum() / s,
                        centerOfMass1.getSum() / s,
                        centerOfMass2.getSum() / s };
}
```

```
  /**
   * special purpose implementation for native byte[] array 4D.
   */
  private final static double[] findCenterOfMass(final byte[] img,
      final int size0, final int size1, final int size2, final int size3)
  {
    final RealSum centerOfMass0 = new RealSum(),
                  centerOfMass1 = new RealSum(),
                  centerOfMass2 = new RealSum(),
                  centerOfMass3 = new RealSum(),
                  sum = new RealSum();

    int i = 0;

    for (int d3 = 0; d3 < size3; ++d3)
      for (int d2 = 0; d2 < size2; ++d2)
        for (int d1 = 0; d1 < size1; ++d1)
          for (int d0 = 0; d0 < size0; ++d0) {
            final double value = img[i++] & 0xff;
            centerOfMass0.add(value * d0);
            centerOfMass1.add(value * d1);
            centerOfMass2.add(value * d2);
            centerOfMass3.add(value * d3);
            sum.add(value);
          }
    final double s = sum.getSum();

    return new double[] { centerOfMass0.getSum() / s,
                          centerOfMass1.getSum() / s,
                          centerOfMass2.getSum() / s,
                          centerOfMass3.getSum() / s };
  }

  /**
   * special purpose implementation for native byte[] array 5D.
   */
  private final static double[] findCenterOfMass(final byte[] img,
      final int size0, final int size1, final int size2, final int size3, final int size4)
  {
    final RealSum centerOfMass0 = new RealSum(),
                  centerOfMass1 = new RealSum(),
                  centerOfMass2 = new RealSum(),
                  centerOfMass3 = new RealSum(),
                  centerOfMass4 = new RealSum(),
                  sum = new RealSum();

    int i = 0;

    for (int d4 = 0; d4 < size4; ++d4)
      for (int d3 = 0; d3 < size3; ++d3)
        for (int d2 = 0; d2 < size2; ++d2)
```

```
          for (int d1 = 0; d1 < size1; ++d1)
            for (int d0 = 0; d0 < size0; ++d0) {
              final double value = img[i++] & 0xff;
              centerOfMass0.add(value * d0);
              centerOfMass1.add(value * d1);
              centerOfMass2.add(value * d2);
              centerOfMass3.add(value * d3);
              centerOfMass4.add(value * d4);
              sum.add(value);
            }
    final double s = sum.getSum();

    return new double[] { centerOfMass0.getSum() / s,
                          centerOfMass1.getSum() / s,
                          centerOfMass2.getSum() / s,
                          centerOfMass3.getSum() / s,
                          centerOfMass4.getSum() / s };
  }

  /**
   * special purpose implementation for native byte[] array 6D.
   */
  private final static double[] findCenterOfMass(final byte[] img,
      final int size0, final int size1, final int size2, final int size3, final int size4,
      final int size5)
  {
    final RealSum centerOfMass0 = new RealSum(),
                  centerOfMass1 = new RealSum(),
                  centerOfMass2 = new RealSum(),
                  centerOfMass3 = new RealSum(),
                  centerOfMass4 = new RealSum(),
                  centerOfMass5 = new RealSum(),
                  sum = new RealSum();

    int i = 0;

    for (int d5 = 0; d5 < size5; ++d5)
      for (int d4 = 0; d4 < size4; ++d4)
        for (int d3 = 0; d3 < size3; ++d3)
          for (int d2 = 0; d2 < size2; ++d2)
            for (int d1 = 0; d1 < size1; ++d1)
              for (int d0 = 0; d0 < size0; ++d0) {
                final double value = img[i++] & 0xff;
                centerOfMass0.add(value * d0);
                centerOfMass1.add(value * d1);
                centerOfMass2.add(value * d2);
                centerOfMass3.add(value * d3);
                centerOfMass4.add(value * d4);
                centerOfMass5.add(value * d5);
                sum.add(value);
              }
    final double s = sum.getSum();
```

```java

    return new double[] { centerOfMass0.getSum() / s,
                          centerOfMass1.getSum() / s,
                          centerOfMass2.getSum() / s,
                          centerOfMass3.getSum() / s,
                          centerOfMass4.getSum() / s,
                          centerOfMass5.getSum() / s };
  }

  /**
   * special purpose implementation for native byte[] arrays 1D to 6D.
   * dispatches to the 1D to 6D versions.
   */
  public final static double[] findCenterOfMass(final byte[] img, final int[] dimensions)
  {
    if (dimensions.length == 1)
      return findCenterOfMass(img, dimensions[0]);
    else if (dimensions.length == 2)
      return findCenterOfMass(img, dimensions[0], dimensions[1]);
    else if (dimensions.length == 3)
      return findCenterOfMass(img, dimensions[0], dimensions[1], dimensions[2]);
    else if (dimensions.length == 4)
      return findCenterOfMass(img, dimensions[0], dimensions[1], dimensions[2],
          dimensions[3]);
    else if (dimensions.length == 5)
      return findCenterOfMass(img, dimensions[0], dimensions[1], dimensions[2],
          dimensions[3], dimensions[4]);
    else if (dimensions.length == 6)
      return findCenterOfMass(img, dimensions[0], dimensions[1], dimensions[2],
          dimensions[3], dimensions[4], dimensions[5]);
    else
      throw new IllegalArgumentException("only 1D to 6D images are supported");
  }
}
```

3.8 Center-Of-Mass for C++

```cpp
#ifndef _CENTER_OF_MASS_H
#define _CENTER_OF_MASS_H

#include <vector>
#include "realsum.hh"

namespace internal
{
  template<int M, typename T, typename S>
  struct sum
  {
    static void apply(long pos[], S sums[], T w)
    {
      sums[M - 1] += w * pos[M - 1];
      sum<M - 1, T, S>::apply(pos, sums, w);
    }
```

```
  };

  template<typename T, typename S>
  struct sum<0, T, S>
  {
    static void apply(long pos[], S sums[], T w) {}
  };

  template<int N, int M, typename T, typename S>
  struct center_of_mass
  {
    static void apply(const std::vector<long>& dimensions, long pos[], S sums[], T*& data)
    {
      for (pos[N - 1] = 0; pos[N - 1] < dimensions[N - 1]; ++pos[N - 1])
        center_of_mass<N - 1, M, T, S>::apply(dimensions, pos, sums, data);
    }
  };

  template<int M, typename T, typename S>
  struct center_of_mass<0, M, T, S>
  {
    static void apply(const std::vector<long>& dimensions, long pos[], S sums[], T*& data)
    {
      sums[M] += *data;
      sum<M, T, S>::apply(pos, sums, *data);
      ++data;
    }
  };
}

template<int N, typename T>
void center_of_mass(const std::vector<long>& dimensions, T* data, double c[])
{
  long pos[N];
  RealSum<double> sums[N+1];
  internal::center_of_mass<N, N, T, RealSum<double> >::apply(dimensions, pos, sums, data);
  for (int d = 0; d < N; ++d)
    c[d] = (double) sums[d] / (double) sums[N];
}

#endif
```

4. GAME OF DEATH

In order to illustrate the level of generality ImgLib2 offers, we developed a program that efficiently simulates the growth and death of life forms in a certain area, the so-called *Arena*. Several life forms grow and fight to become the dominant race.

The simulation seeds the *Arena* with several races of life forms. Each life form has a certain name (i.e. its race) and a weight representing its population at a spot. They grow every round by a certain percentage (e.g. 10%) and die of hunger if the population at a spot becomes too large. Every round each race at each spot tries to spread into their local neighborhood, trying to invade new space. If a neighboring spot is empty they will simply occupy it and start growing. If the same race is present in their vicinity, their population simply adds up.

If another race is present they will fight for the spot. The race with higher population at this spot will survive, however their population will decrease by the amount of population the defeated race had lost.

Using only these parameters, the simulation always converges to a point where one race wins. Therefore, we added the possibility of an epidemic which kills 90% of the dominating race, keeping the entire system in an equilibrium.

In order to simulate this relatively complex behavior, we implemented a new *NumericType* called *LifeForm*. Both operations, growth and fight can be simulated by implementing specialized multiplication and addition methods, respectively (see LifeForm.java). Multiplying a LifeForm with a floating-point value represent growth (or shrinkage in case of an epidemic) while addition of two LifeForms represents the fight for a certain spot. The actual dynamics in the *Arena*, i.e. the spreading and the fight for every spot are a diffusion operation that is very efficiently simulated running the generic implementation of the *Gaussian Convolution*, treating each spot of the *Arena* as a pixel in a *RandomAccessibleInterval* (or simply *Img*).

The process of such a simulation was exemplarily recorded for the first 1000 frames and is available as supplementary movie. The format is divx, we suggest using the Videolan client for playback. The simulation as shown in the supplement ($640 \times 480, \sigma = 2.5$, 5 races) was recorded with 11 frames per second on a 13“ Macbook Pro with 2.7 Ghz Intel Core i7 processor.

The entire source code for the simulation consists of three classes. *Arena.java* is the main class running the simulation, *LifeForm* implements the specialized mathematical operations required for the simulation, and *LifeFormARGBConverter* implements methods to map the state of a Life Form into an ARGB value for display.

4.1 Arena.java

Arena.java is the main class running the simulation. It is possible to change all major parameters of the simulation. An additional class, called *ArenaMovie.java*, is available in the imglib2 repository and is capable of recording movies as shown in the supplement.

```
public class Arena
{
  // a central random number generator
  final static Random rnd = new Random(System.currentTimeMillis());

  // number of seeds for LifeForms
  final int numSeeds = 100000;

  // we simulate with 5 races
  final int numRaces = 5;

  // the overall growth of all races per round
  final float growth = 1.05f;

  // all races above this weight will die of lack of food
  final float maxWeight = 1.1f;

  // chance for a epedemic (in percent)
  final float epidemic = 0.1f;

  // the sigma of the gaussian convolutionm
  // determines how far each race spreads from a spot
  final float sigma = 2.5f;

  // the width and height of the image
  final int width = 640;
```

```java
final int height = 480;

// the out of bounds strategy to use for gaussian convolution
// makes a significant difference to the result
final OutOfBoundsFactory<LifeForm, RandomAccessibleInterval<LifeForm>> outofbounds =
  new OutOfBoundsPeriodicFactory<LifeForm, RandomAccessibleInterval<LifeForm>>();

public Arena()
{
  // create a new ArrayImgFactory for LifeForm
  final ArrayImgFactory<LifeForm> factory = new ArrayImgFactory<LifeForm>();

  // create the ArrayImg containing the simulation
  Img<LifeForm> arena = factory.create(new long[] { width, height }, new LifeForm());

  // seed the arena with a number of random life forms
  seedArena(arena, numSeeds, numRaces);

  // init the display
  final LifeFormARGBConverter display = new LifeFormARGBConverter();

  // show the initial image (will be updated in each step)
  final ImagePlus imp = ImageJFunctions.wrapRGB(arena, display, "Arena");
  imp.show();

  // for computing the frames per second
  final long start = System.currentTimeMillis();
  long numFrames = 0;

  // repeat until cancelled
  while (true)
  {
    // growth of each life form every round
    for (final LifeForm t : arena)
    {
      t.mul(growth);

      // if they grow too much they will die because of lack of food
      if (t.getWeight()> maxWeight)
        t.setWeight(0.001f);
    }

    // simulate diffusion by gaussian convolution
    Gauss.inNumericTypeInPlace(new double[]{ sigma, sigma }, arena, outofbounds);

    // compute and display frames per second
    final double fps = ++numFrames*1000 / (double)(System.currentTimeMillis() - start);
    imp.setTitle("fps: " +
      NumberFormat.getInstance().format(fps) + " frame: " + numFrames);

    // we regularly have an epidemic
    epidemic(arena, epidemic, numRaces);
```

```java

      // update the LifeFormARGBConverter to the current min and max value of the weight
      display.setMin(0);
      display.setMax(getMax(arena));

      // update the ImageJ display to the current state of the simulation
      updateDisplay(imp, arena, display);
    }
  }

  /**
   * Given a certain chance there is an epidemic killing 90% of the dominant race
   *
   * @param arena - the simulation
   * @param chance - the chance of having an epidemic
   * @param numRaces - the number of races
   */
  protected void epidemic(final Img<LifeForm> arena, final float chance, final int numRaces)
  {
    // is there an epedemic?
    if (rnd.nextFloat() * 100 <chance)
    {
      // which race does it hit?
      final int race = dominantLifeForm(arena, numRaces);

      for (final LifeForm l : arena)
        if (l.getName() == race)
          l.mul(0.1f);
    }
  }

  /**
   * Returns which LifeForm is currently dominating
   *
   * @param arena - the simulation
   * @return - index of the dominant LifeForm
   */
  protected int dominantLifeForm(final Img<LifeForm> arena, final int numRaces)
  {
    final double[] countRaces = new double[ numRaces ];

    for (final LifeForm l : arena)
      countRaces[ l.getName() ] += l.getWeight();

    double last = countRaces[ 0 ];
    int race = 0;

    for (int i = 1; i <numRaces; ++i)
      if (countRaces[ i ]> last)
      {
        last = countRaces[ i ];
        race = i;
```

```
      }

    return race;
  }

  /**
   * Seed the arena with a number of random life forms
   *
   * @param arena - the Img containing the Life forms
   * @param numSeeds - the number of seeds
   * @param numRaces - the number of races to use
   */
  protected void seedArena(final Img<LifeForm> arena,
    final int numSeeds, final int numRaces)
  {
    final int numDimensions = arena.numDimensions();
    final RandomAccess<LifeForm> randomAccess = arena.randomAccess();

    for (int i = 0; i <numSeeds; ++i)
    {
      for (int d = 0; d <numDimensions; ++d)
        randomAccess.setPosition(Math.round(rnd.nextFloat() *
          (arena.dimension(d) - 1)), d);

      randomAccess.get().set(i % numRaces, 1);
    }
  }

  /**
   * Compute the maximum weight of all pixels
   *
   * @param img - the Img containing the state of the simulation
   * @return the maximum weight
   */
  protected float getMax(final Img<LifeForm> img)
  {
    float max = img.firstElement().getWeight();

    for (final LifeForm l : img)
      if (l.getWeight()> max)
        max = l.getWeight();

    return max;
  }

  /**
   * Update the ImageJ display window with the current state of the simulation
   *
   * @param imp - the displayed ImagePlus
   * @param img - the Img containing the state of the simulation
   * @param display - the LifeFormARGB converter that can convert a
   *                  LifeForm into an ARGB representation
```

```java
     */
    protected void updateDisplay(final ImagePlus imp, final Img<LifeForm> img,
      final LifeFormARGBConverter display)
    {
      // create a new ImagePlus based on the current state of the simulation
      final ImagePlus impNew = ImageJFunctions.wrapRGB(img, display, "Arena");

      // set the pixels of the new ImagePlus to the already displayed ImagePlus
      imp.getProcessor().setPixels(impNew.getProcessor().getPixels());

      // update the already displayed ImagePlus
      imp.updateAndDraw();
    }

    public static void main(String[] args)
    {
      // init ImageJ Window
      new ImageJ();

      // Start the fight
      new Arena();
    }
```

4.2 LifeForm.java

The *LifeForm* class stores the current state of a life form, i.e. its name and its weight. Furthermore, it implements a subset of the *NumericType* methods (add, mul) that allows to run a Gaussian convolution on a dataset consisting of life forms.

LifeForm implements the interfaces *NumericType* and *NativeType*. The *NumericType* type interface requires implementation of the standard mathematical operation add(), mul(), div() and sub(). This enables to run Gaussian Convolution on a dataset consisting of *LifeForms*. As we only require add() and mul() for the simulation, we simply did not implement div() and sub(). The *NativeType* interface requires the implementation of methods that allow *LifeForm* to act as *proxy type*, which means it can map itself into a Java native array. To store the name and weight we use an native integer-backed array using two integers per pixel. The second int storing the weight is bit-wise converted to a float. Note that the simulation would run as well if it would not implement *NativeType*, however using significantly more memory as each pixel has to be an individual object. Additionally, this requires using a *ListImg* instead of an *ArrayImg* or *CellImg*.

The mathematical operations are implemented as follows:

- add(LifeForm c)
  - if 'c' has the same name, the weight of 'c' will be added to this LifeForm
  - if 'c' has a different name, this LifeForm will take the name of the LifeForm with the higher weight, but the weight of the defeated LifeForm is subtracted
- mul(double c)
  - the weight of this LifeForm is multiplied by 'c'

Running a Gaussian Convolution on such a dataset will simulate the diffusion of each of the LifeForms to its neighboring area.

```java
public class LifeForm implements NumericType<LifeForm>, NativeType<LifeForm>
{
  // the current index of the cursor or randomAccess
  protected int i = 0;

  // the index of name and weight in the current IntAccess
  protected int nameI = 0, weightI = 1;

  // the underlying container holding the data (could be ArrayImg, CellImg, ...)
  final NativeImg<LifeForm, ? extends IntAccess> storage;

  // the current array that holds the information, which one (e.g which cell
  // of a CellImg) it is is defined by the cursor/randomAccess
  IntAccess b;

  // this is the constructor for initializing with an array
  public LifeForm(NativeImg<LifeForm, ? extends IntAccess> lifeFormStorage)
  {
    storage = lifeFormStorage;
  }

  // this is the constructor if you want it to be a variable
  public LifeForm(final int name, final float weight)
  {
    storage = null;
    b = new IntArray(2);

    set(name, Float.floatToIntBits(weight));
  }

  // this is the constructor if you want it to be a variable
  public LifeForm() { this(0, 0); }

  // called by the NativeImgFactory to create a new NativeImg using the type of
  // data the LifeForm requires (in this case an integer array with 2 entries per pixel)
  @Override
  public NativeImg<LifeForm, ? extends IntAccess> createSuitableNativeImg(
    final NativeImgFactory<LifeForm> storageFactory, final long dim[])
  {
    // create the container (int, 2 values per pixel)
    final NativeImg<LifeForm, ? extends IntAccess> container =
      storageFactory.createIntInstance(dim, 2);

    // create a Type that is linked to the container
    final LifeForm linkedType = new LifeForm(container);

    // pass it to the DirectAccessContainer
    container.setLinkedType(linkedType);

    return container;
  }

```

```java
// called by the cursor/randomAccess to update the currently active IntAccess
@Override
public void updateContainer(final Object c)
{
  b = storage.update(c);
}

/**
 * Set the name of this LifeForm
 *
 * @param name - the new name
 */

public void setName(final int name) { b.setValue(nameI, name); }
/**
 * Set the weight of this LifeForm
 *
 * @param weight - the new weight
 */
public void setWeight(final float weight)
{
  b.setValue(weightI, Float.floatToIntBits(weight));
}

/**
 * @return - the name of this LifeForm
 */

public int getName() { return b.getValue(nameI); }
/**
 * @return - the weight of this LifeForm
 */
public float getWeight()
{
  return Float.intBitsToFloat(b.getValue(weightI));
}

/**
 * Set name and weight of this LifeForm
 * @param name - the new name
 * @param weight - the new weight
 */
public void set(final int name, final float weight)
{
  setName(name);
  setWeight(weight);
}

/**
 * @param c - the LifeForm to "add" to this one
 */
@Override
```

```java
    public void add(final LifeForm c)
    {
      final float a = getWeight();
      final float b = c.getWeight();

      final int na = getName();
      final int nb = c.getName();

      if (na == nb)
        setWeight(a + b);
      else
      {
        if (a < b)
          set(nb, b - a);
        else
          setWeight(a - b);
      }
    }

    /**
     * @param c - multiply weight of this LifeForm by 'c'
     */
    @Override
    public void mul(final float c)
    {
      setWeight(getWeight() * c);
    }

    /**
     * @param c - multiply weight of this LifeForm by 'c'
     */
    @Override
    public void mul(final double c)
    {
      setWeight((float)(getWeight() * c));
    }

    /**
     * not required/not defined
     */
    @Override
    public void div(final LifeForm c)
    {
      throw new UnsupportedOperationException("LifeForm.div() is not supported ");
    }

    /**
     * not required/not defined
     */
    @Override
    public void mul(final LifeForm c)
    {
```

```
    throw new UnsupportedOperationException("LifeForm.mul() is not supported ");
  }

  /**
   * not required/not defined
   */
  @Override
  public void sub(final LifeForm c)
  {
    throw new UnsupportedOperationException("LifeForm.sub() is not supported ");
  }

  /**
   * set weight of this LifeForm to 1
   */
  @Override
  public void setOne() { setWeight(1); }

  /**
   * set weight of this LifeForm to 0
   */
  @Override
  public void setZero() { setWeight(0); }

  /**
   * create a new, uninitialized LifeForm
   */
  @Override
  public LifeForm createVariable() { return new LifeForm(); }

  /**
   * for internal use, create a new LifeForm that works on the defined NativeImg
   */
  @Override
  public LifeForm duplicateTypeOnSameNativeImg() { return new LifeForm(storage); }

  /**
   * the currnet index in the IntAccess, definied by the cursor/randomAccess
   */
  @Override
  public int getIndex() { return i; }

  /**
   * Set the values of this LifeForm to the values of LifeForm 'c'
   * @param c - new LifeForm
   */
  @Override
  public void set(final LifeForm c) { set(c.getName(), c.getWeight()); }

  /**
   * @param i - set the index in the IntAccess (called by cursor/randomAccess)
   */
```

```java
	@Override
	public void updateIndex(final int i)
	{
	  this.i = i;
	  nameI = i * 2;
	  weightI = i * 2 + 1;
	}

	/**
	 * increase the index in the IntAccess by one (called by cursor/randomAccess)
	 */
	@Override
	public void incIndex()
	{
	  ++i;
	  nameI += 2;
	  weightI += 2;
	}

	/**
	 * increase the index in the IntAccess by 'increment' (called by cursor/randomAccess)
	 * @param increment
	 */
	@Override
	public void incIndex(final int increment)
	{
	  i += increment;

	  final int inc2 = 2 * increment;
	  nameI += inc2;
	  weightI += inc2;
	}

	/**
	 * decrease the index in the IntAccess by one (called by cursor/randomAccess)
	 */
	@Override
	public void decIndex()
	{
	  --i;
	  nameI -= 2;
	  weightI -= 2;
	}

	/**
	 * decrease the index in the IntAccess by 'increment' (called by cursor/randomAccess)
	 * @param increment
	 */
	@Override
	public void decIndex(final int decrement)
	{
	  i -= decrement;
```

```java
    final int dec2 = 2 * decrement;
    nameI -= dec2;
    weightI -= dec2;
  }

  /**
   * create a String representation of this LifeForm
   */
  @Override
  public String toString()
  {
    return "Race " + getName() + ", Weight " + getWeight();
  }

  /**
   * clone this LifeForm
   */
  @Override
  public LifeForm clone() { return copy(); }

  /**
   * copy this LifeForm
   */
  @Override
  public LifeForm copy(){ return new LifeForm(getName(), getWeight()); }

  /**
   * @return - how many entities per pixel are used
   */
  @Override
  public int getEntitiesPerPixel(){ return 2; }
}
```

4.3 LifeFormARGBConverter.java

LifeFormARGBConverter creates a virtual ARGB representation of a *LifeForm* for display. This is achieved by implementing the interface *Converter*⟨*LifeForm, ARGBType*⟩. Currently, up to seven different races can be mapped into seven different colors for display. Note that the simulation itself supports an arbitrary number of *LifeForms*. The weight of a *LifeForm* will represent its intensity by mapping its float value to a value between 0. . . 255, based on the minimal and maximal weight of the entire *Arena*.

```java
public class LifeFormARGBConverter extends AbstractLinearRange implements
  Converter<LifeForm, ARGBType>
{
  /**
   * Instantiate a new LifeFormARGBConverter where min=0 and max=1
   */
  public LifeFormARGBConverter()
  {
    super();
  }

  /**
```

```java
   * Instantiate a new LifeFormARGBConverter
   * @param min - the minimal weight for display (will map to intensity 0)
   * @param max - the maximal weight for display (will map to intensity 255)
   */
  public LifeFormARGBConverter(final double min, final double max)
  {
    super(min, max);
  }

  /**
   * Convert the LifeForm to an ARGB value
   * @param input - the LifeForm to convert
   * @param output - the ARGBType that will contain the RGB representation
   */
  @Override
  public void convert(final LifeForm input, final ARGBType output)
  {
    final int col = (short)Math.round(normFloat(input.getWeight()) * 255);

    final int name = input.getName();

    if (name == 0)
      output.set(col<<16);
    else if (name == 1)
      output.set(col<<8);
    else if (name == 2)
      output.set(col);
    else if (name == 3)
      output.set((col<<16) + (col<<8));
    else if (name == 4)
      output.set((col<<16) + (col<<8) + col);
    else if (name == 5)
      output.set((col<<16) + col);
    else if (name == 6)
      output.set((col<<8) + col);
  }

  /**
   * norm the weight of the LifeForm to 0...255 using min and max
   * @param c
   * @return
   */
  public float normFloat(final float c)
  {
    double value = (c - min) / (max - min);

    if (value < 0)
      value = 0;
    else if (value > 1)
      value = 1;

    return (float)value;
```

```
    }
}
```

5. CLASS DIAGRAM

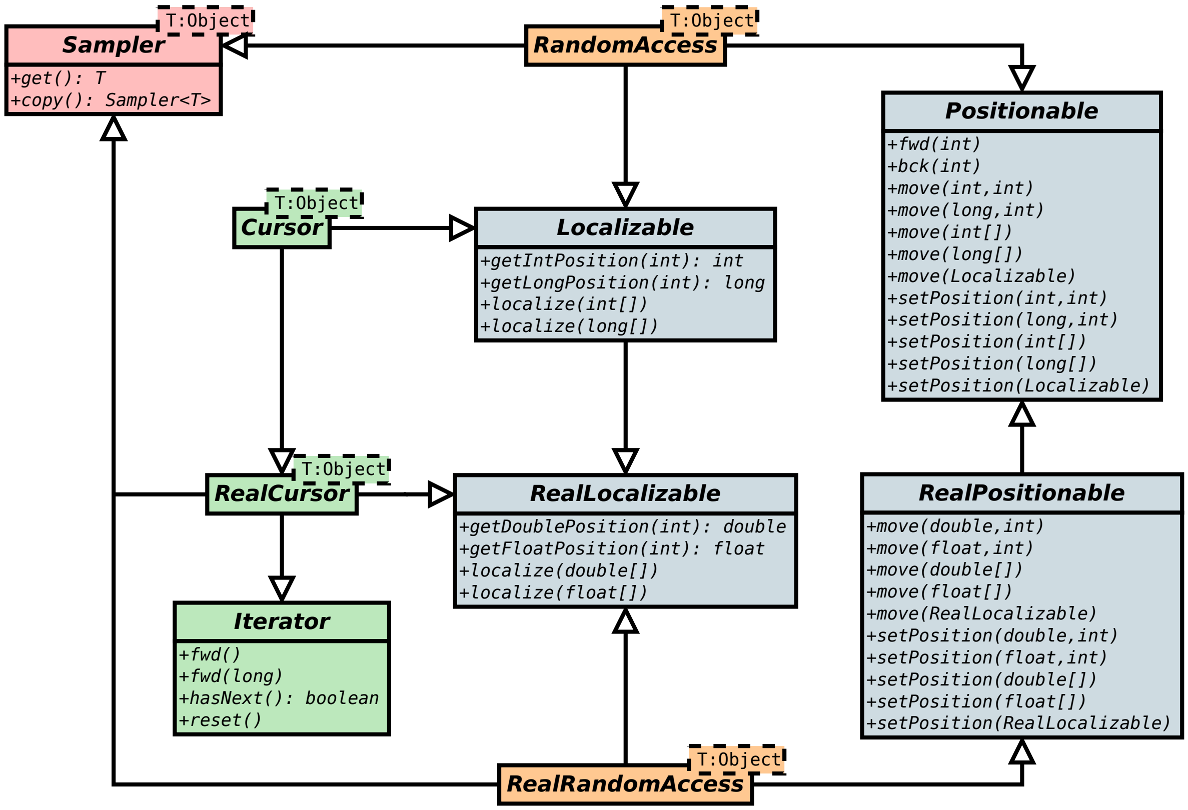

Figure 2. Interfaces for access to pixel values and coordinates. `Sampler<T>` provides access to pixel values from a generic pixel value domain. `Localizable` and `RealLocalizable` provide read access to integer and real precision coordinates. `Positionable` and `RealPositionable` provide write access to integer and real precision coordinates. `RandomAccess` and `RealRandomAccess` combine pixel value access and random coordinate access. With `Iterator`, all pixels can be visited once. The order of traversal is subject to special purpose implementation and expected to minimize access time. `Cursor` and `RealCursor` combine pixel access by iteration and localization. Typically, there are two variants of Iterators available. One that calculates its location per each iteration and one that calculates it only per localization request. The former is more efficient when localization occurs frequently, the latter otherwise.

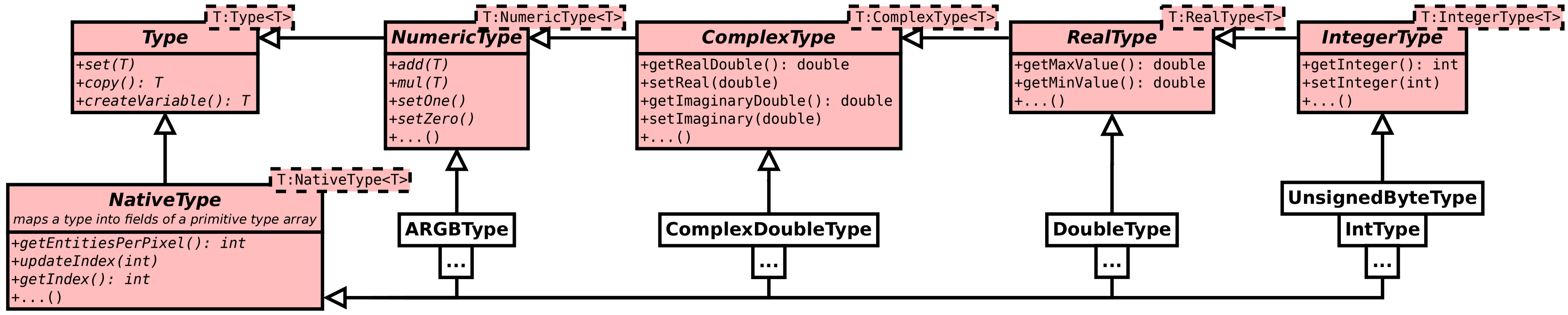

Figure 3. A fragment of the ImgLib2 pixel type hierarchy. Pixel type interfaces serve two purposes: Firstly, they specify a set of algebraic operations to be used for pixel processing. Re-usability of algorithms can be maximized by implementing them using the minimal set of operations required (e.g. addition and multiplication for convolution). Secondly, pixel types that implement the `NativeType<T>` interface can be mapped into an array of Java primitive types. Such proxy types avoid the memory and runtime overhead that would be inevitable when storing individual pixels as Java object instances.

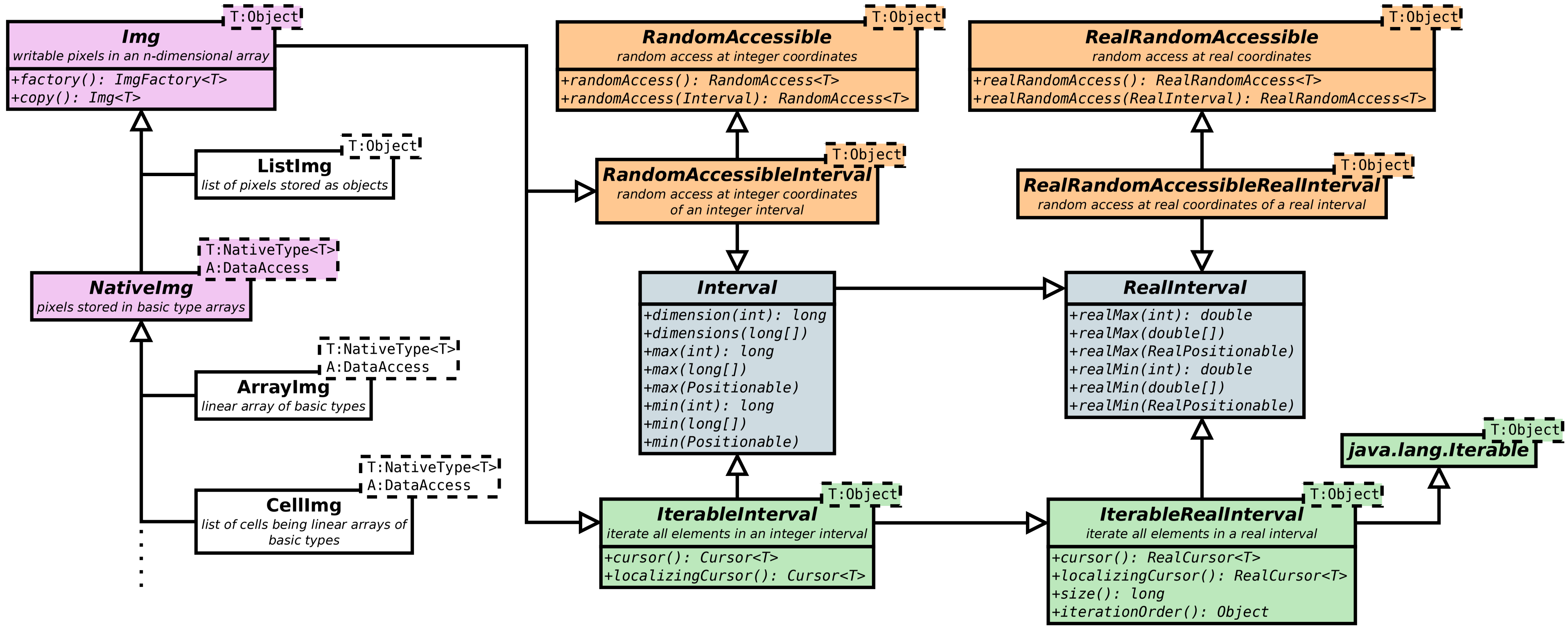

Figure 4. On the right, basic ImgLib2 collections interfaces. An image is a mapping from a subset of n-dimensional Euclidean coordinate space (domain) to a generic pixel Type. We distinguish integer and real precision coordinates, bounded and unbounded, randomly accessible and iterable domains, either continuous or discrete with values arranged on a grid or sparsely sampled. A conventional pixel image is a `RandomAccessibleInterval<T>` and `IterableInterval<T>`: it comprises a bounded grid of pixels of generic Type T that are random-accessible and iterable. Note, that the ImgLib2 image interfaces support many concepts that go beyond the classic pixel image. For example, a continuous `RealRandomAccessible<T>` may be procedurally generated or interpolated from discrete data such as pixel images or sparsely sampled datasets. On the left, exemplary concrete implementations to store n-dimensional pixel data in a regular grid. `ListImg` stores pixels as object instances and can be used for any kind of object but not for large numbers of pixels. `ArrayImg` maps pixels into a single Java primitive type array. `CellImg` splits the coordinate space into a grid of rectangular cells, each mapping pixels into a Java basic type array. For mapping into arrays, `NativeType` pixels are required.
